# Epigenetics of methylation modifications in diabetic cardiomyopathy

**DOI:** 10.3389/fendo.2023.1119765

**Published:** 2023-03-15

**Authors:** Jing Hao, Yao Liu

**Affiliations:** ^1^ Department of Emergency, Children’s Hospital of Nanjing Medical University, Nanjing, China; ^2^ Department of Pharmacy, Children’s Hospital of Nanjing Medical University, Nanjing, China

**Keywords:** T2DM, diabetic cardiomyopathy, histone methylation, DNA methyaltion, epigenetic

## Abstract

Type 2 diabetes is one of the most common metabolic diseases with complications including diabetic cardiomyopathy and atherosclerotic cardiovascular disease. Recently, a growing body of research has revealed that the complex interplay between epigenetic changes and the environmental factors may significantly contribute to the pathogenesis of cardiovascular complications secondary to diabetes. Methylation modifications, including DNA methylation and histone methylation among others, are important in developing diabetic cardiomyopathy. Here we summarized the literatures of studies focusing on the role of DNA methylation, and histone modifications in microvascular complications of diabetes and discussed the mechanism underlying these disorders, to provide the guidance for future research toward an integrated pathophysiology and novel therapeutic strategies to treat or prevent this frequent pathological condition.

## 1 Introduction

According to the International Diabetes Federation Atlas, the prevalence of diabetes is estimated to be 537 million worldwide in 2021 and is dramatically expected to increase to 738 million by 2045 (1). Diabetic cardiomyopathy (DCM) is clinically defined as myocardial disease that occurs in diabetic patients and cannot be explained by coronary artery disease, valvular disease, and other conventional cardiovascular risk factors, including hypertension and dyslipidemia. DCM is manifested by myocardial fibrosis, cardiomyocyte hypertrophy, apoptosis, metabolic dysregulation, and ultimately heart failure (2). Approximately 12% of diabetic patients were affected by DCM, leading to heart failure and death (2). Until now, standard methodology for DCM diagnosis was not developed, possibly because of unrecognized molecular mechanisms. There are more reliable imaging and pathological diagnostic criteria, including decreased left ventricle diastolic dysfunction or left ventricular ejection fraction (EF), or both, pathological left ventricular hypertrophy, and interstitial fibrosis (3). Many previous studies have clearly highlighted the complexity of DCM pathogenesis, and identified the molecular mechanism that synergistically damages cardiomyocytes and the overall function of heart (4). Genetic, dietary, and lifestyle factors are essential in diabetes pathophysiology and associated DCM.

Epigenetics refers to changes in gene expression levels based on non-genetic sequence alterations. Epigenetic modifications, such as DNA methylation, histone modifications, and post-transcriptional RNA regulation, are increasingly considered as important mediators of complex interactions between genes and environmental factors (5–7). This review summarizes the current understanding of epigenetic regulation in type 2 diabetes mellitus (T2DM) and DCM to highlight the impact of DNA and histone methylation in developing of DCM (**Table 1**). Their aberrant regulation is associated with disease progression, etiologically and inducement. Deep investigation and understanding their link may guide future research toward an integrative pathophysiological approach and provide novel therapeutic direction in this field.

**Table 1:** Summary of studies focusing on DNA and histone methylation related to DCM.

Genes	Data sources	Status	References
**AR**	STZ-induced diabetic rat model	Hypermethylation	Tao et al. (2020) (8)
**KEAP1**	T2DM patients	Hypomethylation	Liu et al. (2016) (9)
**p21^WAF1/CIP1^**	cardiac cells (isolated from diabetic patients and STZ-induced diabetic rat model)	Complete methylation	Mönkemann et al. (2002) (10)
**Cyclin D_1_**		Complete demethylation	
**FTO**	T2DM female patients	Hypermethylation	Bell et al. (2010) (11)
**PPARγ**	C57BL/6J mice	Hypermethylation	Yang et al. (2014) (12)
**LXRα**	STZ-induced diabetic rat model	Hypomethylation	Cheng et al.(2011) (13)
**SERCA2a**	HL-1 cells	Hypermethylation	Kao et al. (2010) (14)
**NF-κB**	C57B/6 mice	Hypomethylation	El-Osta et al. (2008) (15)

## 2 DNA methylation modifications and DCM

**Figure 1:**
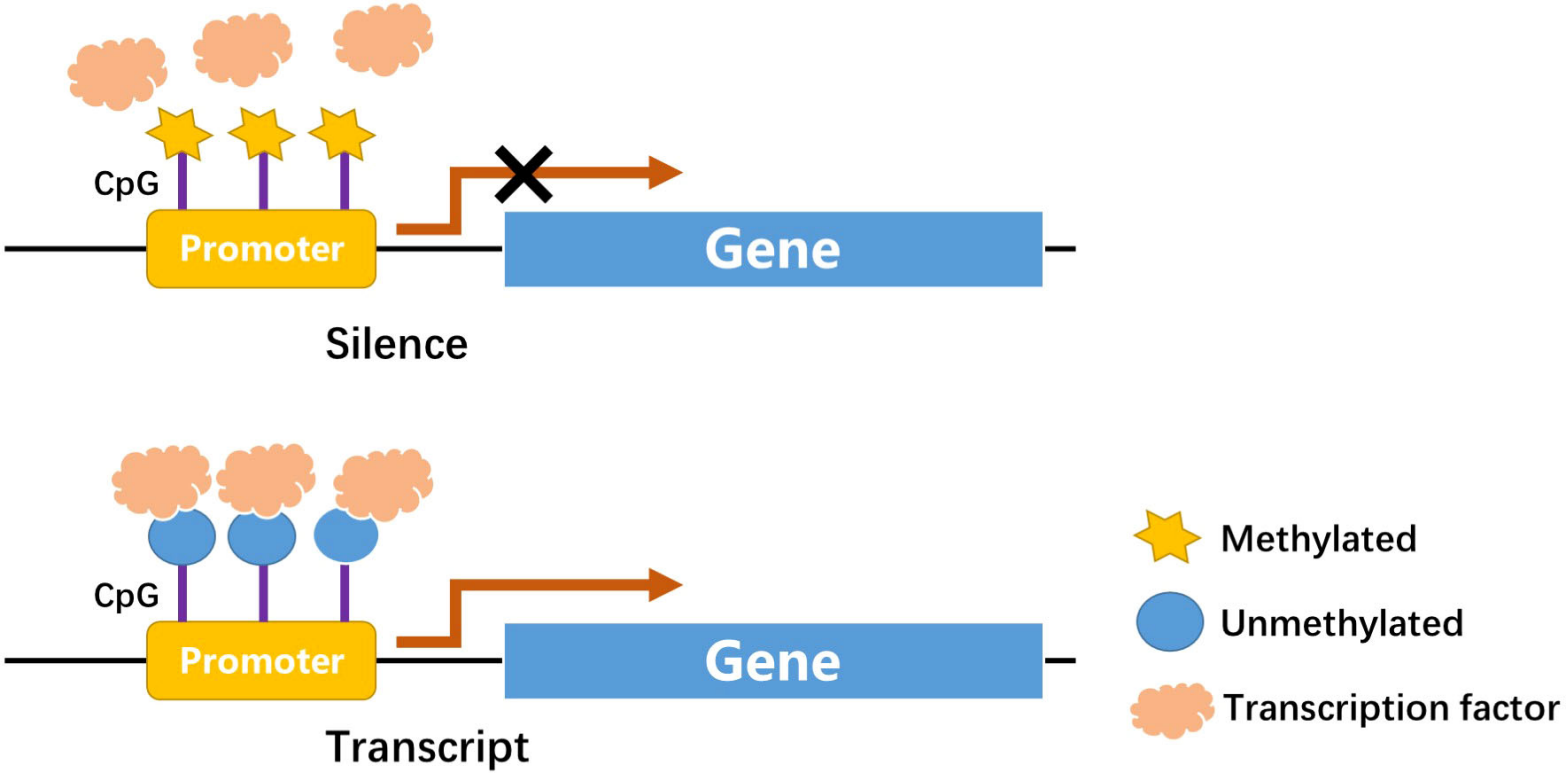
CpGs at promoter site: Methylation in CpGs leads to gene silence.

DNA methylation is one of the first discovered mechanisms of epigenetic regulation, as early as the genetic substance DNA was first discovered (16, 17). In a broad sense, DNA methylation refers to the conversion of specific bases on DNA sequence to S-adenosyl methionine (SAM) under the catalysis of DNA methyltransferase (DNMT) (18). As a methyl donor, the process of chemical modification of a methyl group is obtained through covalent bonding. DNA methylation modification can occur at C-5 position of cytosine and N-6 position of adenine (19). The most frequently methylated nucleotides are located in CpG dinucleotides (20), usually located at the 5’ end of many regulatory gene regions, but can extend into exons. In mammals, CpG exists in two forms: one is dispersed in the DNA sequence, and the other is highly aggregated, called CpG island (CpG island). In normal human cells, 70%–90% of interspersed CpG dinucleotides are modified by methylation (21). CpG islands are often unmethylated (except for some special segments and genes). Research evidence shows that changes in DNA methylation may increase the prevalence of type 1 and type 2 diabetes (22). Abnormal methylation in promoter and other regulatory regions, such as its enhancers, can inhibit transcriptional activity by blocking transcription factors binding to target gene motifs, subsequently causing gene silencing and diseases (**Figure 1**) (22). DNA methylation, one of the most stable epigenetic modifications, is associated with many vital processes and metabolic diseases including obesity, T2DM, and cardiovascular disease (23, 24). DNA methylation status of inflammatory genes, glucose and lipid metabolism genes has been reported to be altered in diabetes (25).

### 2.1 DNA methylation & oxidative stress

Diabetes-induced oxidative stress induced by diabetes is an essential role in diabetes-related cardiovascular complications. In a high glucose environment, continuous oxidation of glucose to mitochondria can trigger massive production of Reactive oxygen species (ROS) and disruption of the oxidation-reduction balance, downregulating the expression of matrix metalloproteinases (MMPs). Dysregulation of the balance between *MMPs* and tissue-type inhibitors of metalloproteinases *(TIMPs) in vivo* is fundamental in pathological processes such as myocardial fibrosis and cardiac remodeling (26). Liu et al. (9)showed significant hypomethylation of *KEAP1* (Kelch-like ECH-associated protein 1) promoter CpG islands in patients with DCM, and elevated KEAP1 protein levels in these patients. *NRF2* activates several antioxidant enzymes. Under normal physiological conditions, NRF2 binds to KEAP1 protein and exists in the cytoplasm in an inactive state, which maintains the low transcriptional activity of *NRF2* by targeting proteases for degradation (27). However, under oxidative stress, the *NRF2*- *KEAP1* interaction dissociates in a dose-dependent manner (28). Liu et al. also reported that the decreased NRF2 antioxidant system in the diabetic heart may change redox homeostasis and lead to aggravated oxidative stress (9). A similar study reported that the gene encoding p21 was overexpressed in the heart of diabetic rats and that the expression of gene encoding cyclin D1was regulated by its 5’ lateral region demethylation and hypermethylation (10). P53-induced alterations in the methylation status of p21WAF1/CIP1 promoter led to the activation of apoptotic pathway, resulting in cardiomyocyte death and cardiomyopathy in rats. The authors proposed that oxidative damage is the main cause of p53-induced p21WAF1/CIP1 gene *de novo* methylation.

### 2.2 DNA methylation & hyperglycemia and insulin resistance

Insulin resistance (IR) and hyperglycemia are fundamental to the onset and DCM development (29, 30). Pirola et al. (31) observed that hyperglycemia significantly affected human vascular chromatin, leading to differential methylation and acetylation patterns associated with transcriptional upregulation of genes related to metabolism and cardiovascular disease. In glucose-treated cells, there was a significant correlation between hyperacetylation and DNA methylation, suggesting that hyperglycemia-induced genes are mediated by changes in methylation and acetylation patterns of genes (32). Additionally, it was reproducibly corroborated the correlation of DNA methylation and cardiovascular risk (11, 33). The susceptibility haplotype rs8050136 of fat mass and obesity associated *(FTO)* gene is a significant gene associated with an increased risk of obesity and CVD with elevated methylation levels (11); a similar mechanism is hypothesized for rs9939609 polymorphism (33). Another candidate gene study found an association between *IGF2* methylation and altered lipid profiles in obese children. Particularly, *IGF2* hypermethylation was associated with a higher triglyceride/HDL-cholesterol ratio, representing an epigenetic marker of metabolic risk (34). Another study examining genome-wide transcriptome and CpG methylation analysis reported many differentially methylated region-predicted loci in adipose tissue from insulin-resistant patients compared to controls, including genes involved in insulin signaling and interactions with integrins (35). Altered methylation was also found in *IL18, CD44, CD48, CD38, Cd37, CX3CL1, CXCR1, CXCR2, CXCL1, IGF1R, APOB48R, LEF1, GIPR, GRB10, SIRT2, HDAC4, DNMT3A, LEPR* and *LEP* genes loci strongly and independently associated with insulin resistance correlated (36–38). Peroxisome proliferator-activated receptors (PPAR) γ, one of three PPAR isotypes, enhances insulin sensitivity, lipogenesis function (39). PPARγ is expressed mostly in adipose tissue and its inactivation could lead to lipodystrophy and metabolic disorder (40, 41). Furthermore, polarization of adipose tissue macrophages from anti-inflammatory to pro-inflammatory phenotype in obese mice involved methylation of *PPARγ* promoter (12).

### 2.3 DNA methylation & myocardial hypertrophy

Liver X receptors (LXRs) are ligand-activated transcription factors belonging to the nuclear hormone receptor superfamily (42–44). LXR, especially LXRα, can regulate the transcription of the gene by binding to the LXR response element (LXRE) on the target gene, thereby regulating cholesterol metabolism and lipid metabolism (45). LXRα and its downstream regulatory network are involved in developing DCM. Previous studies found significant methylation of LXRα gene promoter region in rats with DCM (13). Cheng et al. (46) found that in a streptozotocin-induced diabetic rat model, demethylation of LXRα was responsible for its increased expression in the myocardial ventricles of diabetic rats.

Another study showed that tumor necrosis factor (TNF)-α increased DNMT levels, which enhanced methylation of the sarco/endoplasmic reticulum Ca^2+^-ATPase 2a (*SERCA2a*) promoter region, thereby decreasing SERCA2a expression in cardiac myocytes (14). SERCA2a mediates the heart relaxation by transferring Ca2+ from the cells into the sarcoplasmic reticulum. Downregulation of SERCA2a expression can cause diastolic dysfunction and ultimately lead to the development of DCM (47). The angiotensin II receptor gene in the renin-angiotensin-aldosterone (RAAS) system is divided into two subtypes, AT1 and AT2 (48). AT1 is further divided into two subtypes, AT1a and AT1b (49). Experimental studies in humans and animals have found that AT1 receptor gene polymorphisms are strongly correlated with essential hypertension and corresponding end-organ damage (50). The overexpression of AT1 receptor can cause heart disease and increase the response to Ang II (51). Bogdarina et al. (52), demonstrated that the proximal promoter of AT(1b) gene in the adrenal gland, which expression highly depends on promoter methylation *in vitro*, is significantly undermethylated, and *ATR* genes pathway are upregulated in DCM, leading to cardiac hypertrophy. These relevant data suggest that the expression of RRAS related genes is regulated by DNA methylation with different directions of methylation expression and may play essential roles in DCM pathogenesis.

### 2.4 DNA methylation & ventricular dysfunction

Prolonged high glucose toxicity and multiple factors can induce overexpression of numerous cytokines, increased extracellular matrix production and deposition in the myocardial interstitium and perivascular areas, decreased myocardial compliance, and induced simultaneous ventricular diastolic dysfunction in late stages (2, 53, 54). Different promoter methylation has been reported in DCM. Movassagh et al. (55) characterized DNA methylation profiles in left ventricular tissue from patients with idiopathic and end-stage heart failure. They observed an increase promoter methylation of three genes associated with cardiac angiogenesis in cardiomyopathy, *PECAM1*, *ARHGAP24*, and *AMOTL2*, suggesting that DNA methylation induces altered gene expression in cardiomyopathy (55). However, the pattern of DNA methylation seen in the diabetic heart differs from that in patients with heart failure (56). The specific DNA methylation CpG site ofβ-myosin heavy chain (*β-MYH7*) gene was found to be extensively methylated in T2DM hearts compared to the three spots in the control and heart failure groups. Similar DNA methylation changes were observed in T1DM hearts. In steroid-induced diabetic hearts altered DNA methylation of specific CpG sites of *β-MYH7* might lead to ventricular dysfunction in diabetic patients (56).

### 2.5 DNA methylation & myocardial fibrosis

Early manifestation of DCM is progressive diastolic heart failure, manifested by decreased myocardial relaxation and increased stiffness, with interstitial fibrosis of the myocardium as the primary pathophysiological mechanism. Tao H et al. concluded that DNMT1 inhibition or knockdown increased androgen receptor (AR) expression in cardiac fibroblasts. In addition, AR was found to negatively regulate homocysteine (Hcy)-induced autophagy in cardiac fibroblasts. DNMT1 was shown to enhanced cardiac fibroblast autophagy in diabetic cardiac fibrosis by inhibiting AR axis. DNMT1 inactivation in AR axis triggers cardiac fibroblast autophagy in diabetic cardiac fibrosis (8). Another study showed that DNMT3A directly inhibits miR200b expression, which promotes cardiac autophagy and ultimately affects the development of myocardial fibrosis in a mouse model of aortic constriction (57). MiR3695p overexpression directly inhibits DNMT3A, induces aberrant DNA methylation of Patched1, and suppresses cardiac fibroblast proliferation and myocardial fibrosis levels (58). Therefore, DNA methylation-related modifying enzymes affect fibrosis-related genes expression and regulate myocardial fibrosis through direct or indirect mechanistic pathways. El-Ostaet al. (15) reported short-term exposure of aortic endothelial cells to high glucose-induced promoter DNA methylation of NF- κ B p65 subunit, an essential mediator of cardiac fibrosis. The authors showed that DNA methylation was mediated by a hyperglycemia-induced increase in methylglyoxal production.

## 3 Histone methylation modification and DCM

Histone methylation modifications are among the most studied histone modifications based on the methylation of terminal N atom of lysine or arginine residues (59). Lysine residues are capable of mono-, di-, and trimethylation (me1, me2, me3), whereas arginine residues are only capable of mono- and dimethylation, and these different levels of methylation significantly increase the complexity of histone methylation modifications and regulation of gene expression. Histone methylation modifications have been shown to be essential for the transcriptional regulation of genes, maintenance of genomic integrity, epigenetic modifications. Methylation modification of particular histone lysine or arginine residues is associated with gene activation or repression (60, 61). The role of histone methylation in DCM has received increasing attention. Through the interaction of histone methyltransferases and demethylases, histone methylation plays an equally important role in abnormal glycolipid metabolism, cardiomyocyte hypertrophy, extensive myocardial fibrosis, and cardiac diastolic and systolic dysfunction caused by DCM, which will provide new ideas and therapeutic targets for the study and treatment of DCM (62).

### 3.1 Histone methylation modification & insulin resistance

Euchromatic histone lysine methyltransferase 2 (EHMT2, also known as G9a), located on euchromatin, mediates the methylation of H3K9 and H3K27, leading to gene silencing (63). It has been shown that G9a can affect insulin receptors (IRs) transcription, a key regulator of insulin receptor gene transcription, *via* the high mobility group AT-hook1 (HMGA1). HMGA1 expression level regulates insulin signaling in the liver, and restoring the expression level of G9a in leptin receptor gene-deficient mice not only elevates HMGA1 level, but also reduces hyperglycemia and hyperinsulinemia. Thus, improving the impaired insulin signaling in the liver, and G9a is expected to be a potential therapeutic target for hepatic IR (13). Human homolog of drosophila zeste gene enhancer 2 (EZH2), a subunit of the polycomb repressive complex 2 (PRC2), mainly mediates H3K27me2/3 methylation. High glucose concentrations elevate EZH2 expression *via* the JNK/Notch pathway, which further causes histone H3K27me2/3 upstream of the insulin gene promoter, thereby suppressing insulin gene expression and causing blood glucose elevation, leading to IR (64).

### 3.2 Histone methylation modification & cardiac lipid deposition

When free fatty acid (FFA) metabolism is activated, fatty acid β-oxidation in diabetic myocardium increases and excess FFA can be converted to triglycerides and accumulate in myocardial cells, accelerating lipid deposition and gradually damaging myocardial calmodulin, which in turn affects diastolic and systolic functions of myocardial cells (65). PPARαwill retard FFA metabolism and increase lipid accumulation in cardiomyocytes (66). Lipotoxicity is a fundamental factor for DCM pathogenesis. Increased lipids promote lipid uptake by cells in the vascular wall, and very low-density lipoproteins are more readily converted to cholesteryl esters. Glycated low-density lipoproteins impair its recognition by hepatocyte receptors and slow down its metabolism. It is preferentially phagocytosed and degraded by macrophages through binding to other receptors, accumulating in macrophages as foam cells and eventually presenting with widespread, diffuse myocardial damage associated with metabolism. Cardiomyocyte hypertrophy, degeneration, focal necrosis, and replacement of necrotic areas by fibrous tissue severely affect the cardiomyocyte metabolism, which in turn affects the diastolic and systolic functions of the heart (67).

Histone lysine methyltransferases are mainly composed of SET (su var 3-9, ez, trithorax) structural domain family and the non-SET structural domain family. Among them, SET domain bifurcated histone lysine methyltransferase 1 catalyzes the trimethylation of histone H3K9, and its accumulation decreases the expression of PPARγ and CCAAT-enhancer binding protein α genes and target genes related to lipid metabolism, inhibiting the lipid accumulation in adipocytes (68). There is a lack of literature demonstrating that histone methylation modifications can directly regulate the expression of PPARγ in cardiac muscle tissue. However, histone methylation has been shown in adipose tissue to modify the expression of some critical genes, involving *PPARγ*, *PPARα*, and *IGF2*, to affect obesity progression (69). Knockdown of H3K36 methyltransferase *NSD2* (nuclear receptor binding set domain protein 2) can be observed as a PPARγ-dependent impairment of adipogenesis. Therefore, it is feasible to investigate DCM pathogenesis in terms of histone methylation modifications affecting lipid deposition in the heart.

### 3.3 Histone methylation modification & inflammatory immunity

T2DM leads to adipose tissue dysfunction, the persistent secretion of inflammatory cytokines and chemokines that induce chronic low-grade inflammation throughout the body. Chronic high glucose stimulation induces secretion of inflammatory factors that affect the balance between MMPs and TIMPs, leading to rearrangement of collagen molecular structure, which in turn affects collagen synthesis and degradation in the extracellular matrix. Thickening of myocardial fibers and increased myocardial stiffness decrease compliance and diastolic function, constituting the structural basis for impaired cardiac function (70, 71). Nuclear factor kappa-B (NF-κB) signaling pathway is one of the most important signaling pathways in the inflammatory response. Its activation promotes the expression of multiple pro-inflammatory factors, chemokines, and adhesion molecules (72). Under the stimulus of persistent hyperglycemia *in vivo* in diabetic vascular complications, NF-κB protein expression is regulated by histone methylation level, and its reduced expression is closely associated with increased H3K4me1 and decreased H3K9me2/me3 in the promoter region of subunit p65 (73). Genome-wide association analysis showed that altered intracellular levels of H3K4me2 and H3K9me2 in human monocytes treated with high glucose directly affected gene expression, including interleukin (IL)-1 and IL-8 (74).

Diabetes mellitus is an etiological risk for coronary heart disease, and diabetes develop chronic and persistent inflammation and cardiovascular complications even after stabilized glycemic control, suggesting a possible “metabolic memory” (75, 76). A cytological experiment showed a strong link between chromatin histone methylation and metabolic memory. Compared to normal cultured cardiomyocytes, the level of IL-6 mRNA levelwas increased in cardiomyocytes cultured with high glucose. Simultaneously, the protein level of suppressor of variegation 3-9 homolog 1 (Suv39h1), a methyltransferase homolog of H3K9me3, was reduced by high glucose treatment, and H3K9me3 level in the IL-6 promoter region was significantly lower. Therefore, regulation of histone methylation and inflammatory cytokine expression may be an effective strategy to prevent metabolic memory and cardiomyopathy in diabetic patients (76).

### 3.4 Histone methylation modification & oxidative stress

Studies have confirmed that histone methylation modifications are closely associated with antioxidant synthesis and peroxide scavenging *in vivo (* 77). Up-regulation of H3K4 dimethylation levels in the catalytic subunit of glutamate cysteine ligase (catalytic, Gclc) antioxidant response element region of the antioxidant glutathione synthase reduced glutathione synthesis; this result was reversed by interfering with cells using siRNA for the specific demethylase LSD1 (78). Elevated levels of H4K20 trimethylation on the manganese-containing superoxide dismutase (MnSOD) gene SOD2 promoter caused a decrease in MnSOD expression but increased intracellular ROS levels (79). SET8 is the enzyme that monomethylates H4K20, and overexpression of SET8 reduces the accumulation of ROS and restores NO levels, thereby reducing inflammation in vascular endothelial cells (80). Reducing the functional impairment of endothelial cells induced by high glucose damage during diabetes-induced cardiovascular disease (81).

### 3.5 Histone methylation modification & autophagy

Autophagy is a highly conserved phagocytic degradation process in living organisms, which is closely related to the metabolic needs of cell survival and renewal of some organelles (82). Autophagy is essential in maintaining cellular homeostasis and is closely associated with disorders of glucolipid metabolism, enhanced oxidative stress, IR, and deposition of advanced glycation end products in DCM patients (83). Recent studies have shown that histone methyltransferases are involved in the transcriptional regulation of autophagy and regulators of various biological processes (84). When the organism is starved, glucose supply is insufficient and/or exposed to rapamycin, the di-methylation of arginine residues is significantly increased, leading to autophagy. H3K9me2, dependent on histone methylation transferase G9a has been shown to achieve the protective effect of distal ischemic preadaptation by regulating expression of mammalian targets of the rapamycin complex (85). Coactivator-associated arginine methyltransferase 1 can increase the level of H3K17me2 and activate the expression of autophagy and lysosome-related genes (86). Meanwhile, histone demethylase LSD1 can negatively regulate the autophagic process in cardiomyocytes by promoting PTEN degradation (87).

## 4 Pharmacological intervention and clinical practice

### 4.1 A potential biomarker

The early stage of T2DM is usually ignored because of no symptoms, but microvascular and macrovascular complications may have occurred during this period or even in pre-diabetic patients (88). Therefore, in terms of treatment, finding reliable, sensitive and easily accessible T2DM biomarkers is helpful for early diagnosis, treatment and management of patients. Epigenetic changes in insulin target organs can be reflected in blood, DNA methylation in blood may be related to the occurrence of T2DM, and related differentially methylated genes can be used as potential candidate biomarkers of T2DM (89, 90). With the advancement of technologies related to human genomics, the focus of epigenetic research has shifted to high-throughput epigenome-wide association study (EWAS), such as microarray chips and methylation sequencing technologies.

#### 4.1.1 T2DM-related differentially methylated genes discovered based on microarray chip methylation analysis

Microarray chip-based methylation analysis is help in quantifying the methylation level of DNA samples by comparing the hybridization intensity ratio of DNA samples before and after digestion with methylation-sensitive restriction endonucleases (91). It is the earliest EWAS technique to analyze genome-wide methylation changes associated with T2DM (92). Toperoff et al. (93) compared DNA methylation levels in peripheral blood of 710 T2DM patients and 459 healthy individuals based on microarray chip technology, and found that an excess of differential methylation sites in genomic regions which previously confirmed genes related to T2DM, among which the most differentially methylated sites are located in the following genes: Solute carrier family 30 member 8 (*SLC30A8*), transcription factor 7-like 2 (transcription factor 7-like 2, *TCF7L2*), fat mass and obesity associated (*FTO*) gene, and potassium channel protein 1 (potassium voltage-gated channel subfamily Q member 1, *KCNQ1*) gene. In the next step, they found a CpG site in the first intron of *FTO* gene that showed significant hypomethylation in T2DM patients compared to the healthy volunteers. This result was also confirmed in Arab population by similar procedures (94). These studies revealed that low methylation levels at specific sites in T2DM-associated genomic regions would be a warning marker of T2DM.

#### 4.1.2 T2DM-related differentially methylated genes discovered based on microbead array chip methylation analysis

Bead array chip-based DNA methylation analysis aims to provide single-base resolution analysis and quantitative evaluation of specific cytosines in multiple samples. Chambers et al (95) analyzed DNA methylation associated with T2DM in the whole peripheral blood of Indian, Asians, and Europeans using human methylation 450 microbead array chips. They followed them up with patients who eventually developed T2DM. The result showed methylation markers at five loci including thioredoxin-interacting protein (*TXNIP*), ATP-binding cassette subfamily G(*ABCG1*), phosphoethanolamine/phosphocholine phosphatase (*PHOSPHO1)*, suppressor of cytokine signaling 3(*SOCS3*) and sterol regulatory element binding transcription factor 1(SREBF1) were related to the increased risk for T2DM. Subsequently, Dayeh et al. (96)evaluated the sites identified by Chambers et al., and confirmed an association between the methylation levels of ATP-binding cassette subfamily G1 (*ABCG1)* and Phosphoethanolamine/Phosphocholine phosphatase (*PHOSPHO1)* gene-related sites in whole blood DNA and the risk of developing T2DM. Relationship between the methylation status of ABCG1 gene-related sites and the fasting blood glucose and insulin levels was also confirmed in other studies using microbead array chips (97, 98). Al Muftah et al (99) quantified DNA methylation in whole blood DNA from Arab subjects, and verified CpG sites of 8 genes associated with the risk of T2DM, including *TXNIP*. Their result showed that *TXNIP* gene methylation was suppressed in T2DM patients. Moreover, TXNIP can participate in the development of T2DM-related vascular complications by inhibiting the ability of vascular endothelial growth factors to regulate angiogenesis (100–102).

### 4.2 A novel therapeutic target

Mitchel Tate et al. proposed that DNA metalation may play an important role in DCM and thereby represent a potential therapeutic target (103). Unfortunately, their paper does not discuss the specifics of DNA methylation as a target for DCM therapy.

The expression of tissue specific insulin genes is partially regulated by DNA methylation, and the demethylation of insulin promoter CpG is crucial for in the maturation of β-cells (104). There is evidence that transcriptional coactivator peroxisome proliferator-activated receptor gamma coactivator-alpha (protein PGC-1α, gene PPARGC1A) mRNA expression is reduced in islets from T2DM patients and could be regulated by DNA methylation of PPARGC1A promoter (105). In summary, the expression of genes related to IR and DCM could be regulated by DNA methylation, leading to impaired systolic function, increased oxidative stress, cardiac remodeling and cardiomyocyte apoptosis. Therefore, regulating DNA methylation to inhibit IR may be a promising new direction for early blood glucose control and prevention of DCM progression.

Zhou et al. recently reported that flavonoids can be used as natural epigenetic modulators for DCM management (106). They discussed the epigenetic effects of different flavonoid subtypes in DCM and summarized the existing evidence from preclinical and clinical studies. For example, quercetin, which is an antioxidant, reduced oxidative stress by preventing the decrease in GSH/GSSG ratio, NRF2 nuclear translocation, and antioxidant enzymatic activity. Quercetin also prevents a reduction in ATP levels and alterations in PGC-1α, UCP2, and PPARγ expression, leading to improved DCM (107, 108). Epigallocatechin-3-gallate, another flavonoid with antioxidant effects, attenuates oxidative stress by decreasing the methylation status of the *Klotho* gene promoter under high-glucose conditions in diabetic db/db mice and HK-2 cells (109). Genistein, a soybean isoflavone, can inhibit DNA methylation and histone acetylation in addition to reduce renal fibrosis in unilateral ureteral occlusion mice (110). Interestingly, it recently reported that genistein could attenuate marijuana-induced vascular inflammation (111). These two reports suggest that regulating DNA methylation status may be another possible pathway for genistein to exert the cardiovascular protective effect. However, the bioavailability of flavonoid compounds is relatively low, and the current research is mainly focused on cell and animal experiments, which shows a long way from bench to bed.

Furthermore, some recent experimental studies have focused on Epi-drugs in DCM treatment. Zhu H et al. found that AGE exposure increased the expression of *DNMT1* and *DNMT2*, resulting in decreased expression and activity of GPX1 in the heart. Supplementation of selenium preparations can recover the expression of *DNMT2* and restore the expression and activity of GPX1, which could alleviate intracellular ROS generation and cardiomyocyte apoptosis, and lead to recovery of cardiac function. Selenium supplementation or administration of the DNMT inhibitor AZA decreased DNA methylation at the promoter of GPX1 gene (112). Kakoki M et al. found that feeding superoxide scavenger cyanocobalamin (B12) prevented and reversed signs of cardiomyopathy in type 1 diabetic Elmo1H/H Ins2Akita/+ mice. Diabetes significantly reduced plasmaIGF-1 levels, whereas B12 restored them through DNA methylation of S-adenosylmethionine levels, DNMT-1/3a/3b mRNA, and suppressing cytokine signaling (SOCS)-1/3 promoters normalization to activate hepatic IGF-1 production, and reductions in cardiac IGF-1 mRNA and phosphorylated IGF-1 receptors were also restored, predicting B12 as a promising potential treatment for DCM (113).

## 5 Summary and prospect

**Figure 2:**
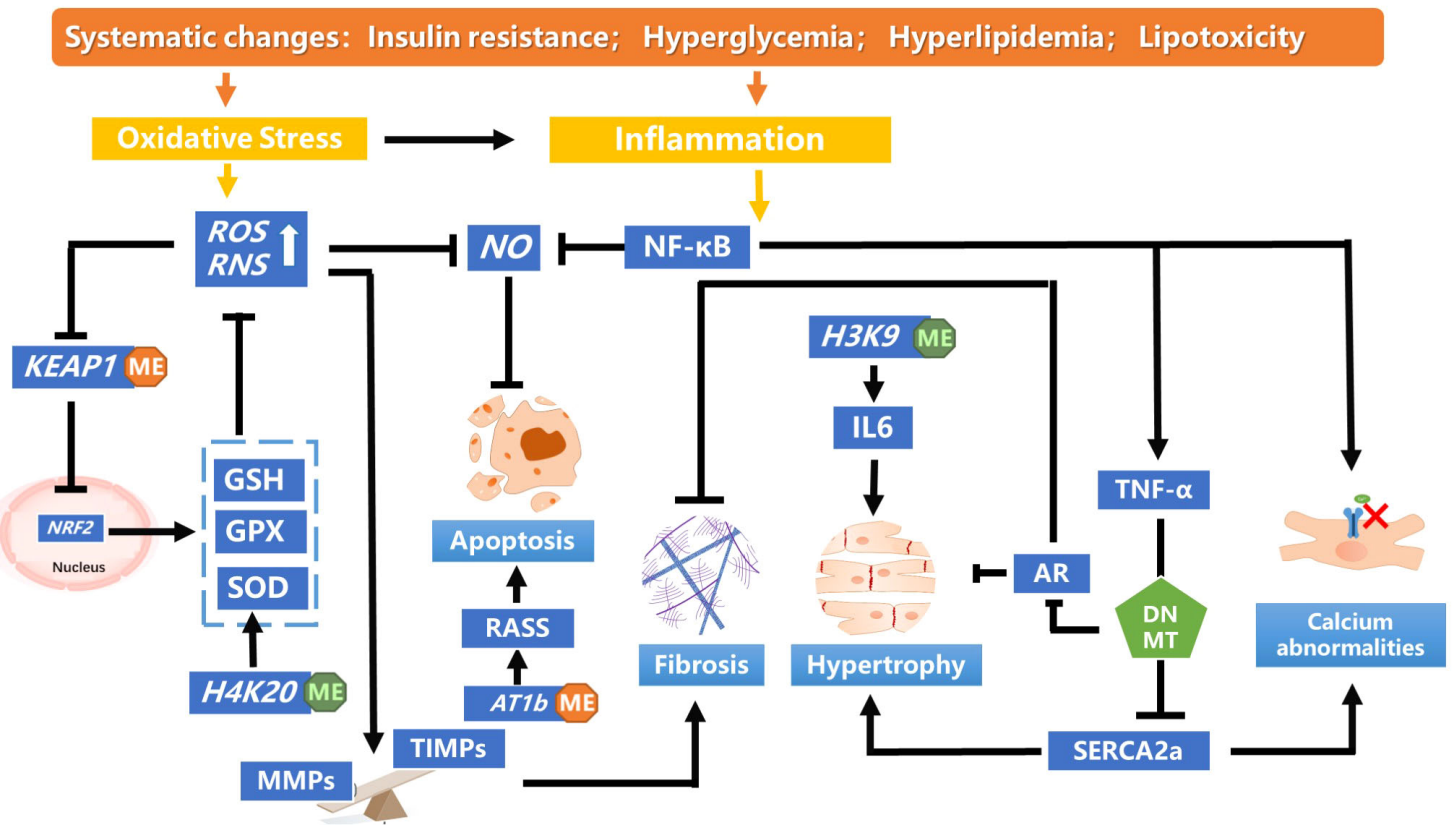
A summary of the DNA methylation and histone modification pathways leading to pathological cardiac dysfunction in diabetic cardiomyopathy. In diabetics, insulin resistance mediates systemic hyperglycemia, hyperlipidemia and lipotoxicity, inducing oxidative stress and inflammation., Hypomethylation of *KEAP1* promoter was observed in patients with DCM, which maintains the low transcriptional activity of *NRF2* by targeting proteases for degradation. The decreased NRF2 antioxidant system changes redox homeostasis and leads to aggravated oxidative stress. Monomethylating H4K20 reduces the accumulation of ROS and restores NO levels, which alleviates oxidative stress. Massive production of ROS disturbs the balance between MMPs and TIMPs, inducing myocardial fibrosis and cardiac remodeling. TNF-α increases DNMT levels, which enhanced methylation of *SERCA2a* promoter, thereby decreasing SERCA2a expression. SERCA2a mediates the heart relaxation by transferring Ca^2+^ from the cells into the sarcoplasmic reticulum. Downregulation of SERCA2a expression cause myocardial hypertrophy. DNMT could enhanced cardiac fibroblast autophagy in diabetic cardiac fibrosis by inhibiting AR axis. Decreased H3K9me3 level in the IL-6 promoter increased its mRNA level, which affect the pathology of myocardial fibrosis and cardiomyocyte hypertrophy. IL, interleukin; NF-κB, nuclear factor-enhanced light chain activator of B cells; NO, nitric oxide; Reactive oxygen species; RAAS, renin–angiotensin–aldosterone system; TNF- α,tumour necrosis factor-alpha.

Metabolic disorders, including diabetes are due to a cumulative interaction of genetic and environmental factors. These effects are mainly caused by diabetes-related factors involving hyperglycemia, oxidative stress, inflammation, and obesity, manifesting as genomic epigenetic changes. DNA methylation and histone modification, are the primary epigenetic modifications associated with DCM (**Figure 2**). They are responsible for altering gene expression of key regulatory pathways mediating diabetes-related vascular complications and are significant contributors to diabetes-related metabolic memory. Herein, we have separately explored the association of DNA methylation and histone methylation with DCM, but our summary of these mechanisms is the tips of iceberg and awaits translational application. Further research focused on elucidating the mechanisms may still be needed.

A range of new tools and technologies (e.g., RNA seq, transcriptomics, metabolomics, epigenomic profiling, and chromatin 3D mapping) have been integrated into diabetes research to gain a deeper understanding of T2DM and its associated microvascular complications. Tissue- and cell-specific analysis of methylation levels and histone modifications of significant pathophysiological genes will increase our understanding of the pathology and associated complications of T2DM. There is also a need to elucidate the association between epigenetic regulation of the genome involved in microvascular complications and macrovascular complications of diabetes. Knowledge gained through altered epigenetic gene expression in DCM will provide a better approach to mitigate hyperglycemia-induced damage to the heart and other affected organs, such as the kidney and brain.

## Author contributions

JH and YL conceptualized and designed the study, carried out the analyses, drafted the initial manuscript, and contributed to the important intellectual content during manuscript drafting and revision. YL contributed to the important intellectual content during manuscript drafting and revision, reviewed and revised the manuscript. 
